# Effects of medical school selection on student motivation: a PhD thesis report

**DOI:** 10.1007/s40037-017-0398-1

**Published:** 2017-12-18

**Authors:** Anouk Wouters

**Affiliations:** 1 Research in Education, VUmc School of Medical Sciences, Amsterdam, The Netherlands; 2 LEARN! research institute for learning and education, Faculty of Psychology and Education, VU University Amsterdam, Amsterdam, The Netherlands

**Keywords:** Selection, Admissions, Motivation, Undergraduate medical education, Self-determination theory

## Abstract

**Introduction:**

High stakes are involved in student selection, for both medical schools and applicants. This thesis investigated the effects of selection on the medical student population and applicant pool in the Dutch setting.

**Methods:**

This thesis consists of six papers: two quantitative studies, one qualitative study, two mixed methods studies and one perspective paper based on a review of the literature.

**Results:**

(1) Compared with a lottery, selection does not result in a student population with better motivation, engagement and performance, both in the clinical and pre-clinical phases of the study. (2) Selection seems to have a temporary stimulating effect on student motivation through enhancing perceived autonomy, competence and relatedness. (3) Applicants adopt a strategic approach, based on the selection procedure, in their choice of medical school. (4) The description of an applicant’s motivation is not a reliable and valid tool to assess motivation during selection. (5) Gaining healthcare experience is crucial for applicants’ motivation, but inequalities in access to such experiences can demotivate certain student groups from applying to medical school. (6) The gains yielded from selection compared with a lottery seem to be small. Unintentionally induced self-selection among certain groups of students and biased selection procedures may compromise student diversity.

**Discussion:**

The added value of selection procedures compared with a weighted lottery for admitting students to medical school is questionable. Students are generally motivated and perform well, irrespective of how they enrolled in medical school. Selection yields only small gains, while student diversity may be hampered.

## Introduction

Several challenges are faced by medical school selection committees who are responsible for admitting the students they expect to successfully complete medical programs and become good doctors [1]. Admissions decisions should be based on selection processes that consist of appropriate selection tools and are equitable to all students. As a compromise between a process that provides equal opportunity (straight lottery selection) and one that rewards achievement (qualitative comparative selection), the Dutch Government introduced a lottery weighted for pre-university grade point average (GPA) in the 1970s [2]. A small number of top pre-university GPA students, with a GPA of ≥8/10, were granted direct access, while for other students the numbers of lottery tickets available were higher for those with higher grade point averages (i. e., 3, 4, 6 and 9 tickets for students with a GPA of <6.5, 6.5–6.9, 7.0–7.4 and 7.5–7.9, respectively). The weighted lottery was gradually replaced with qualitative selection procedures. During this transition period, students who were rejected in selection automatically enrolled in the weighted lottery procedure [3]. The proportion of students admitted through selection increased from 10% in 2000 to 100% in 2017. This transition period created research opportunities for comparing the effects of these different processes.

Research on selection mainly focuses on predicting academic performance [4]. This thesis also explores selection from the perspective of student motivation, as motivation is an important factor in student performance, as well as student learning and well-being. Self-determination theory (SDT) [5] was used as the theoretical framework, as it considers motivation as dynamic and postulates that motivation can be influenced by the educational environment [6]. This allowed selection and motivation to be explored as mutually influencing factors and investigation of the underlying mechanisms. SDT posits that motivation can change from autonomous motivation, which reflects interest in and the acknowledgement of the importance of an activity, to controlled motivation, which reflects internal and external pressure and rewards, and vice versa. SDT research has shown that the fulfilment of three psychological needs, autonomy, competence and relatedness, stimulates autonomous motivation. While autonomous motivation is associated with positive student outcomes such as deep learning, positive well-being and good performance, controlled motivation is associated with poor student outcomes such as exhaustion [6, 7].

The effect of selection on the applicant pool is an important aspect of this thesis, as certain population groups, such as first-generation university students, students from ethnic minority backgrounds and students without parents in the medical profession are underrepresented in medical education [8, 9]. This can be explained partly by biases in selection, but may also be due to self-selection, in which prospective applicants decide whether to apply to medical school based on their knowledge of the medical program or selection.

The main research question of this thesis was:

How does selection affect the motivation of the student population and applicant pool?

## Methods and results

A multi-method approach was applied, in which quantitative findings were obtained through validated questionnaires with good reliability and further explored using qualitative research methods. This approach allowed examining of the mechanisms involved in selection. Power analyses were conducted for each quantitative study. A focused review of the literature formed the basis for a perspective paper.

*Study 1 *[10]: In this cross-sectional, mixed methods, questionnaire-based study among first- and fourth-year students (*n* = 357) in a six-year regular medical program and first-year students in a graduate entry program, we examined the effect of selection on the student population and motivation. We used validated questionnaires, namely the Strength of Motivation for Medical School-Revised (SMMS-R) questionnaire [11] and the Learning Self-Regulation Questionnaire [12] to measure the quantity and quality of motivation for the medical study, respectively. We found higher strength of motivation among selected students than non-selected students (*p* < 0.01), but no differences in the quality of motivation. Recently selected students reported higher strength of motivation (*p* < 0.01), autonomous motivation (*p* < 0.01) and controlled motivation (*p* < 0.05) than students who were selected longer ago and non-selected students. Open questions revealed that students’ needs for autonomy, competence and relatedness were fulfilled by being selected.

*Study 2 *[13]: In this quantitative survey study, we investigated the associations of various admissions processes (i. e. selection, lottery and direct access) and participation in a voluntary selection procedure with student performance, motivation and engagement. We measured the quantity and quality of motivation, and engagement using validated questionnaires, namely the SMMS-R scale [11], Academic Self-Regulation Scale (SRQ-A) [14], and Utrecht Work Engagement Scale-Student (UWES-S-9) [15], respectively. Participants were first- and fourth-year students (*n* = 666) from three medical schools. Top pre-university-GPA students outperformed the other students (*p* < 0.1 and *p* < 0.01). Regression analyses showed highest strength of motivation among Year-1 selected students (*p* < 0.05), but no differences in the quality of motivation and engagement between admission groups. Participation in selection was associated with higher engagement (*p* < 0.05) and better clerkship performance in Year-4 students (*p* < 0.01).

*Study 3 *[16]: In this quantitative survey study, we explored the relation between students’ main reasons for applying to a specific medical school and their motivation during medical school. We measured the quantity and quality of motivation using the SMMS-R [11] and SRQ-A [13], respectively. Participants were first- and fourth-year students (*n* = 478) from three medical schools. Most students had strategically chosen the medical school they applied to, i. e. based on their chances to ace the selection procedure (56.9% and 46.9%), while a minority had made their choice based on the curriculum (11.2% and 12.4%). The different approaches were not related to differences in student motivation during medical school.

*Study 4 *[17]: To investigate whether a written statement on motivation can be used to distinguish between applicants in selection, we carried out a thematic analysis of 96 descriptions of motivation made by medical school applicants. The validity and reliability of written statements to assess motivation in selection are questionable. Applicants seemed to provide similar, socially desirable responses about their motivation, in which controlled motivation appeared to be underreported. Applicants also seemed to use their statements as a means to show their suitability for medical school, which was outside the scope of the assignment.

*Study 5 *[18]: To explore how individuals develop the motivation to pursue medical education and how selection affects the motivation of prospective applicants from various backgrounds, we conducted semi-structured interviews with three high school study counsellors and 24 high school students. The reasons for aspiring for the medical profession mainly pertain to autonomous motivation, specifically to scientific interest and helping people. Students also have reasons pertaining to controlled motivation, such as prestige and a high salary. Exposure to healthcare, which is often one of the selection criteria, seems to be a crucial factor for developing autonomous motivation for studying medicine and helps in making an informed choice. Difficulties in gaining healthcare experience, often due to the lack of a network in the medical profession, can demotivate applicants from choosing to study medicine.

*Paper 6* [19]: A focused literature review provided a holistic perspective on the issue of selection and lottery, indicating that the gains yielded from selection versus a lottery seem to be small. Furthermore, selection may compromise student diversity due to (unintentionally) induced self-selection among underrepresented students in medical education and biased selection procedures.

## Discussion

This thesis raises questions about the added value of expensive and time-consuming selection procedures compared with a lottery [19]. While selection is aimed at increasing the quality of the student population, the findings from the research to date indicate that selection yields only small gains compared with a lottery procedure. The lack of significant findings may be due to a ceiling effect, meaning that all students score near the top on our outcome measures. Student motivation, performance and engagement in the Netherlands can be considered good, irrespective of how students are admitted to medical programs. Efforts to further improve the academic success rates of medical students might be focused more on ensuring that students receive proper training during their six years of medical education rather than on selection. Hubbeling argued that most applicants are likely to be able to complete their studies and become capable doctors if properly trained [20]. Indeed, dropout rates among medical students in the Netherlands are low, and selection results only in a small decrease in these rates [19]. In the research in this thesis, dropout rates were too low to conduct further analyses. Until a reliable and valid method for identifying unsuitable applicants at a young age (i. e. 17–18 years), based on their non-academic personal qualities, is identified, a lottery could be the fairest, most efficient and cheaper alternative [21]. The findings in this thesis indicate that selection stimulates strategic behaviour in applicants. To reach the desired match between student and curriculum, medical schools could use this approach to their advantage by aligning the selection procedure with their curriculum. This can involve including lectures and tests that are representative of the medical curriculum. An issue of concern is that inequality in access to resources relevant for the selection process may compromise the desired student diversity. Students without parents in the medical profession may perceive their chances of success in selection to be lower than those with parents in the medical profession because they have fewer means to prepare for selection. This can negatively affect their motivation and subsequently cause them to refrain from applying. These inequalities need attention if medical schools wish to ensure that the medical profession reflects the ever-increasing diversity in the society it serves [22].

The findings support the following recommendations. Medical schools should review the cost-effectiveness of their selection procedures. Next to financial costs, the negative effects on student diversity need to be considered. If gains in terms of improved performance, motivation and well-being of the student population can be expected to be low, selection could be replaced by a lottery. If selection prevents non-traditional potential applicants from applying to study medicine, measures should be taken against inequalities in admissions and to improve the student diversity. These will be context-dependent and could include creating equal opportunities for all students to acquire healthcare internships and organizing pre-med weeks for underrepresented students. We do not recommend the use of a statement on motivation to assess applicants’ motivation. In fact, every assessment of motivation in selection should be questioned, as applicants are likely to ‘fake good behaviour’. It can, however, serve as a matching tool to encourage applicants to become informed about the medical course. A limitation of this thesis is that the conclusions are based on cross-sectional data. Longitudinal research could reveal how the different admission groups develop throughout the medical study.

### Advice

Your PhD is a learning trajectory. Make use of this educational playground and take the chance to learn about different research methodologies and methods. In addition, take the opportunity to learn from others; both experienced researchers and fellow PhD students. But above all, enjoy this special time of your career.

## University information

The defence took place at the VU University Amsterdam, the Netherlands, on 9 February 2017. The supervisors were Professor Gerda Croiset and Dr. Rashmi A. Kusurkar. This thesis is available online via the university library at http://hdl.handle.net/1871/55083. A PDF version is available upon request from the author.
